# Multilocus Heterozygosity and Coronary Heart Disease: Nested Case-Control Studies in Men and Women

**DOI:** 10.1371/journal.pone.0124847

**Published:** 2015-05-13

**Authors:** Kenneth J. Mukamal, Majken K. Jensen, Tune H. Pers, Jennifer K. Pai, Peter Kraft, Eric B. Rimm

**Affiliations:** 1 Department of Medicine, Beth Israel Deaconess Medical Center, Boston, Massachusetts, United States of America; 2 Department of Nutrition, Harvard School of Public Health, Boston, Massachusetts, United States of America; 3 Center for Biological Sequence Analysis, Technical University of Denmark, Lyngby, Denmark; 4 Division of Genetics, Children's Hospital, Boston, Massachusetts, United States of America; 5 Division of Endocrinology, Children's Hospital, Boston, Massachusetts, United States of America; 6 Medical and Population Genetics, Broad Institute, Cambridge, Massachusetts, United States of America; 7 Institute of Preventive Medicine, Copenhagen University Hospital, Copenhagen, Denmark; 8 Channing Division of Network Medicine, Department of Medicine, Brigham and Women's Hospital and Harvard Medical School, Boston, Massachusetts, United States of America; 9 Department of Epidemiology, Harvard School of Public Health, Boston, Massachusetts, United States of America; 10 Department of Biostatistics, Harvard School of Public Health, Boston, Massachusetts, United States of America; Centro Cardiologico Monzino IRCCS, ITALY

## Abstract

**Background:**

Generalized allelic heterozygosity has been proposed to improve reproductive fitness and has been associated with higher blood pressure, but its association with chronic disease is not well characterized.

**Methods:**

Using the Affymetrix Genome-Wide Human 6.0 array, we performed whole genome scans in parallel case-control studies of coronary heart disease (CHD) nested in the Health Professionals Follow-up Study and Nurses’ Health Study. We examined ~700,000 single nucleotide polymorphisms (SNPs) in 435 men with incident CHD and 878 matched controls and 435 women with incident CHD with 931 matched controls. We examined the relationship of genome-wide heterozygosity with risk of incident of CHD and with baseline levels of cardiovascular risk factors.

**Results:**

In both cohorts, approximately 227650 (SD 2000) SNPs were heterozygous. The number of heterozygous SNPs was not related to risk of CHD in either men or women (adjusted odds ratios per 2000 heterozygous SNPs 1.01 [95% confidence interval, 0.91-1.13] in women and 0.94 [0.84-1.06] in men). We also found no consistent associations of genome-wide heterozygosity with levels of lipids, inflammatory markers, adhesion molecules, homocysteine, adiponectin, or body-mass index.

**Conclusions:**

In these parallel nested case-control studies, we found no relationship of multilocus heterozygosity with risk of CHD or its major risk factors. Studies in other populations are needed to rule out associations with lower levels of heterozygosity.

## Introduction

Although individual humans share some 99.6% of base pairs with others,[1] the size of the human genome dictates that there are nonetheless some 24 million base pairs that differ between individuals. This degree of variation offers substantial opportunity for the manifestation of heterozygote advantage, the phenomenon of biological advantage of mixed over pure breeds.

Multilocus heterozygosity may act in two ways. First, heterozygosity at individual loci may provide an advantage relative to homozygote wild-type or variant alleles, sometimes referred to as overdominance. The best known is variation in genes related to erythrocyte function, many of which confer resistance to malaria.[2] In this setting, homozygotes are disadvantaged by either susceptibility to malaria (for wild-type alleles) or clinical syndromes as sickle cell anemia, thallasemia, and glucose-6-phosphate dehydrogenase deficiency. There is also evidence that heterozygosity at specific loci may be associated with perceived attractiveness of humans to members of the opposite sex.[3,4] Second, heterozygosity as a manifestation of outbreeding reduces risk of homozygosity in disadvantageous alleles, leading in the most adverse situations to recessive diseases.

An important caveat in studies of human heterozygosity is the limited evidence that heterosis confers protection against chronic disease, which is less likely to exert selection pressure than do acute infectious diseases or attractiveness to mates, yet is the dominant cause of mortality in developed nations. Heterozygosity at selected loci has been associated with extreme longevity, but these have not been replicated and it is uncertain that they exemplify generalized heterosis.[5,6] In a genome-wide comparison of populations in the Dalmation Islands,[7] Campbell and colleagues found that overall heterozygosity across populations was associated with lower blood pressure and total cholesterol, but individual heterozygosity was not measured, raising the strong possibility that this reflects population stratification. Among individuals in the Framingham Heart Study, multilocus heterozygosity at 706 single nucleotide polymorphisms (SNPs) was associated with higher blood pressure, but not with several other cardiovascular traits measured simultaneously.[8] To our knowledge, no genome-wide assessment among individuals has evaluated whether heterozygosity confers an advantage on common chronic diseases among adults.

To elucidate whether heterozygote advantage extends to coronary heart disease (CHD), the most common cause of death in the United States,[9] we determined the association of variation in genome-wide single nucleotide polymorphisms (SNPs) with both risk of incident CHD and with several CHD risk factors in two well-characterized parallel cohorts of men and women.

## Materials and Methods

### Study Population and Design

The NHS cohort was established in 1976. The study population consists of 121,700 married female registered nurses aged 30 to 55 years residing in one of 11 larger US states. Women have received follow-up questionnaires biennially to update information on exposures and newly diagnosed illnesses. Since 1980, participants have updated information on diet, alcohol, and vitamin supplements through a food frequency questionnaire every four years.

The HPFS began in 1986, when 51,529 male health professionals 40 to 75 years of age completed the initial 6-page HPFS questionnaire. The population includes 29,683 dentists, 3,745 optometrists, 2,218 osteopathic physicians, 4,185 pharmacists, 1,600 podiatrists, and 10,098 veterinarians. Biennial follow-up has mirrored the NHS.

### Case-Control Sampling

We used data from nested case-control studies of CHD in the NHS and HPFS. Blood samples were requested from all active participants and collected from 32,826 NHS members in 1989–1990 and 18,225 HPFS members in 1993–1994. With the exception of a modestly lower prevalence of smoking, those who returned blood samples did not differ substantially from those who did not in both cohorts; mean ages at blood collection were 64 years in men and 60 in women. Participants underwent local phlebotomy and returned samples to our laboratory via overnight courier. Upon arrival, whole blood samples were centrifuged and stored in cryotubes as plasma, buffy coat, and red blood cells in the vapor phase of liquid nitrogen freezers.

From within these subcohorts of women and men who provided blood samples, we conducted parallel nested case-control studies of CHD, defined as non-fatal myocardial infarction or fatal CHD. We wrote to participants who reported incident CHD on the follow-up questionnaires to confirm the report and request permission to review medical records. We also sought medical records for deceased participants, whose deaths were identified by families and postal officials and through the National Death Index. Physicians blinded to the participant’s questionnaire reports reviewed all medical records. Cases of myocardial infarction and fatal CHD were confirmed through review of medical records, as previously described.[10,11]

In the NHS, we matched women free of cardiovascular disease or cancer in 1990 who sustained an incident MI or fatal CHD through 2004 to two randomly selected controls on the basis of age and smoking using risk-set sampling.[12] In the HPFS, we matched men free of cardiovascular disease in 1994 who developed incident CHD through 2004 to 878 control men. In this study design, a control for an early case may be included again if the person develops CHD during follow-up; after counting such converters only once, the total number of samples sent for genotyping were 1524 women and 1354 men.

### Genotyping

In 2008, DNA was extracted for genotyping at Merck Research Laboratories, North Wales, PA, using the Affymetrix Genome-Wide Human 6.0 array. Quality control criteria used to define unsuccessful genotyping were a call rate <97%, sex mismatch, and Hardy-Weinberg equilibrium p<10^–4^ in controls. Analyses based on principal components were conducted to assess self-reported race. Subsequent analysis sample was restricted to subjects of European ancestry. Self-reported "white" samples with substantial similarity to non-European reference samples (the HapMap Yoruba or Asian samples) were excluded. Three eigenvectors were included as covariates to adjust for potential population stratification in the final sample.

Because women with diabetes were genotyped earlier than those without using slightly different platforms, we excluded the ~28,000 SNPs that were not common to the platforms of both diabetic and non-diabetic women. This left a total of 692,794 SNPs for analysis in women and 724,881 in men.

### Measurement of Risk Factor Levels

All biomarkers were measured on samples stored at -130°C and have been found to be largely unaffected by transport conditions and reproducible within persons over time.[13,14] Measurements included lipids (triglycerides, HDL, calculated LDL), inflammatory markers (C-reactive protein, interleukin-6, fibrinogen), homocysteine, adhesion molecules (E-selectin, intercellular adhesion molecule [ICAM]-1, vascular cell adhesion molecule [VCAM]-1) and total and high molecular weight (HMW) adiponectin. Of these, E-selectin, HMW adiponectin, and homocysteine were only measured in women, and IL-6, fibrinogen, homocysteine, E-selectin, ICAM-1, and VCAM-1 were measured only in the initial subset of case-control pairs among women. We also included body-mass index (BMI) based upon self-reported height and weight, which have been found to be reliable within these cohorts.[15]

### Statistical Methods

We examined the associations of multilocus heterozygosity with CHD and its risk factors in multiple ways. Because the number of SNPs and their associated genes were identical across genotyped individuals, we used both the overall number of heterozygous loci and the proportion of loci that were heterozygous; the latter yielded essentially identical results and are not shown here. We conducted three SNP-level analyses, evaluating all autosomal SNPs, a subset of those within the boundaries of all human protein-coding genes and their 70kb upstream and 20kb upstream flanking regions (n = 562,289), and only nonsynonymous SNPs (n = 4,760). Gene coordinates and nonsynonymous SNPs were retrieved from the Ensembl database version 56.37a.

For incident CHD, we performed unconditional logistic regression with adjustment for matching factors in all analyses; this approach yielded results similar to conditional logistic regression with more consistent model convergence. We report odds ratios per 2000 SNPs for analyses of all SNPs and SNPs within genes and per 25 SNPs for nonsynonymous SNPs; these units reflect ~1 standard deviation in each measure.

Analyses of risk factors were conducted with mixed models, including both cases and controls and adjusting for age, smoking, eigenvectors, and case-control status; this approach provides unbiased estimates when genetic risk factors are not strongly associated with case-control status.[16] Information on allelic dosage was extracted for all SNPs using PLINK,[17] and statistical analysis were performed in SAS version 9 (SAS Institute Inc., Cary, NC).

### Ethics Statement

The study protocol, including genotyping, was approved by the institutional review boards of the Brigham and Women’s Hospital and the Harvard School of Public Health. The completion of the self-administered questionnaire was considered to imply informed consent; participants who provided blood samples additionally provided written informed consent.

## Results

Following cleaning of genotyping data, we included 435 women with incident CHD and 931 control women and 435 men with incident CHD and 878 control men. Table 1 demonstrates the associations of genome-wide heterozygosity with risk of CHD among women and men. In both sexes, we observed no relationships of CHD with overall number of heterozygous loci, number of heterozygous loci within genes, or number of heterozygous nonsynonymous loci. These results were essentially identical when the proportions of heterozygous SNPs overall, within genes, or at nonsynonymous loci were used, or when we restricted to individuals below 65 years of age at blood draw.

**Table 1:** Mean (±SD) numbers of heterozygous loci among CHD cases and controls and odds ratios (95% confidence interval) for CHD associated with heterozygous loci.

	Women			Men		
	Cases (n = 435)	Controls (n = 931)	p-value	Cases (n = 435)	Controls (n = 878)	p-value
Number of heterozygous loci	227650 ±1869	227642 ±2240	0.94	227349 ±2184	227451 ±1860	0.40
Number of heterozygous loci within genes	141601 ±1314	141623 ±1471	0.78	142581 ±1452	142633 ±1276	0.53
Number of heterozygous nonsynonymous SNPs	894 ±27	895 ±27	0.58	914 ±27	912 ±27	0.23
**Odds ratio of CHD per:**						
2000 heterozyg. loci	1.01 (0.91–1.13)	Referent		0.94 (0.84–1.06)	Referent	
2000 heterozygous loci within genes	0.99 (0.84–1.17)	Referent		0.94 (0.79–1.12)	Referent	
25 heterozygous nonsynonymous SNPs	0.97 (0.87–1.08)	Referent		1.07 (0.96–1.19)	Referent	

Odds ratios adjusted for age, smoking, and population eigenvectors.

Table 2 shows the relationship of heterozygosity with levels of selected CHD risk factors. There were only two modestly significant relationships in men (LDL among all SNPs and SNPs within genes and fibrinogen among nonsynonymous SNPs; p = 0.03–0.04) and none that were consistent in both sexes, regardless of which measure of heterozygosity was used. One possible exception was the association of overall heterozygosity and heterozygosity within genes with E-selectin (p = 0.03), which was only measured in women and hence could not be confirmed or refuted in men.

**Table 2:** Association of CHD risk factors with overall heterozygous loci, loci within genes, and nonsynonymous SNPs.

		Women			Men		
	Women/Men	All	Within Genes	Nonsynonymous	All	Within Genes	Nonsynonymous
HDL (mg/dL)	1198/1287	-0.7 ±0.4	-0.9 ±0.6	-0.7 ±0.4	0.3 ±0.3	0.5 ±0.5	-0.08 ±0.3
LDL (mg/dL)	1181/1286	0.3 ±1.0	0.9 ±1.5	1.0 ±1.0	-1.9 ±0.9	-2.7 ± 1.3	-0.6 ±0.8
Triglycerides (mg/dL)	1131/1287	1 ±2	2 ±3	3 ±2	1 ±3	3 ±4	-2 ±3
CRP (mg/dL)	1185/1287	-0.02 ±0.02	-0.01 ±0.02	-0.03 ±0.02	-0.01 ±0.02	-0.01 ±0.02	0.001 ±0.01
IL-6 (pg/mL)	616/762	-0.4 ±0.4	-0.5 ±0.5	0.03 ±0.4	0.03 ±0.5	0.3 ±0.7	0.5 ±0.4
Fibrinogen (mg/dL)	211/762	-0.2 ±7	3 ±10	-2 ±5	-2 ±3	-3 ±4	5 ±3
Homocysteine (μmol/L)	644	-0.1 ±0.2	-0.07 ±0.3	-0.03 ±0.2			
E-selectin (ng/mL)	640	1.5 ±0.8	2.7 ±1.2	0.4 ±0.8			
ICAM (ng/mL)	211/762	-0.8 ±8	-2 ±11	4 ±6	-2 ±4	-0.1 ±5	-4 ±3
VCAM (ng/mL)	209/762	5 ±11	-0.3 ±16	-3 ±8	0.4 ±11	-4 ±17	-4 ±10
Total adiponectin (ng/mL)	1209/762	-20 ±106	-54 ±159	117 ±107	-194 ±318	-94 ±465	-532 ±290
HMW adiponectin (ng/mL)	1209	-3 ±85	1 ±126	85 ±85			
BMI (kg/m^2^)	1323/1304	0.1 ±0.1	0.2 ±0.2	0.1 ±0.1	0.2 ±0.1	0.2 ±0.1	0.02 ±0.08

All estimates adjusted for age, smoking, population eigenvectors, and case-control status.

## Discussion

In this analysis of two prospective case-control studies, we found no consistent evidence of heterozygosity advantage in risk for CHD. The number or proportion of heterozygous loci was unrelated to either incident CHD or to circulating CHD risk factors.

The hypothesis of overdominance associated with heterozygosity at individual loci is attractive and has clear support in a few well-described instances. Certainly, the best recognized of these is the selection pressure exerted by P. falciparum, which infects erythrocytes. The widespread prevalence of falciparum malaria appears to have selected for hemoglobin variants that, while disadvantageous on their own, appear to mitigate the consequences of plasmodial infection.[2] Another proposed manifestation of overdominance may exist in perceived attractiveness, even in non-human primate species.[18] For example, in one study, women rated the faces of men heterozygous at three major histocompatibility complex loci as more attractive than the faces of homozygotes, regardless of the level of genetic similarity between female raters and male subjects.[19]

At the same time, evidence that generalized heterozygosity provides an advantage for risk of chronic disease, and particularly for CHD, is sparse. The Framingham Heart Study reported a positive association of heterozygosity at 706 loci with blood pressure and two related echocardiographic parameters among approximately 1000 participants, but no association with any circulating risk factors or with BMI; no association with risk of CHD *per se* was tested.[8] Because we did not have standardized blood pressure or echocardiographic measurement in our cohorts, we cannot directly replicate their findings, but our results do confirm the lack of association with a large number of putative risk factors and extend these results to clinical CHD endpoints. Why blood pressure might differ from other risk factors in its association with heterosis is necessarily speculative, but it is striking that the observed association in Framingham was positive—that is, greater heterozygosity conferred a phenotype of *higher* blood pressure that might well be protective in the setting of acute primordial stressors like trauma or dehydration, but could be harmful for conditions like CHD that manifest in late adulthood. Thus, we find no clear evidence that multilocus heterozygosity is associated with a lower risk of CHD, the most common cause of mortality in developed nations. Its associations with cancer, dementia, and other chronic diseases remain to be tested, although recent work suggests it may influence schizophrenia.[20]

We found few significant relationships—about the number expected from chance alone—and unsurprisingly these generally did not appear consistent across cohorts. The exception of E-selectin is interesting, as its genetic variation is strongly related to ABO blood group genotype[21] and hence could also be subjected to plasmodial selection pressure.[22] However, there appears to be little ancestral selection pressure in erythrocyte traits among populations of European ancestry and hence even this possible association clearly requires confirmation in other cohorts.

Two differences of our study with more common GWAS designs warrant mention. First, although we performed standard chip-based genotyping as in other GWAS designs, we used a nested case-control design for efficiency within the very large parent cohorts. This has the advantage of preserving power to detect associations with CHD with efficiency and produces unbiased and reasonably precise estimates of relative risk comparable to what would be achieved with a GWAS of the full cohorts of NHS or HPFS (because we genotyped all of the cases that would have been present in the full cohort). While power to examine associations on biomarkers was not as efficiently maintained (because the case-control design explicitly tests a single case definition or endpoint), even those associations had ample precision in our analyses because of their continuous distribution.

Second, we did not conduct an unbiased effort to identify single, specific loci that influence risk of CHD or its risk factors, as occurs in most GWAS. Rather, we tested a specific, global hypothesis about the benefit of widespread variation across the genome. Had our hypothesis been proven correct, the natural next step would be to search for specific loci at which that hypothesis was borne out, more similar to standard GWAS efforts. As a consequence of our single underlying hypothesis, however, our statistical analyses use conventional levels of statistical significance and not the extreme thresholds more familiar in GWAS.

Our study has strengths, but important limitations as well. In both studies, participants who provided blood were middle aged or older, and hence we did not study cases of CHD that occurred early in life, which may be most closely associated with genetic factors, although our findings in those below 65 years of age were also null. We also studied a geographically diverse sample but occupationally homogeneous sample, in whom ascertainment of CHD is reliable but who may not generalize readily to the full US population. We performed genome-wide scans, and hence had an unbiased and comprehensive assessment of variation in SNPs, but we cannot exclude the possibility that heterozygosity at specific loci (e.g., major histocompatibility complex) or in other forms of genetic variation (e.g., copy number variants) favorably influence CHD or its risk factors. Likewise, we studied CHD and its risk factors but cannot rule out associations of heterozygosity with other chronic diseases nor with overall survival.

Power is an important concern in studies of heterozygosity. Number or proportion of heterozygous SNPs are an imperfect estimate of inbreeding,[23] potentially requiring large samples to overcome. Further, we were limited to case-control samples of moderate size with narrow ranges in heterozygosity, illustrated by the small standard deviation in number of heterozygous loci relative to the number genotyped. Nonetheless, our point estimates demonstrated no association whatsoever with CHD risk, and our study compares favorably in size to the Framingham Heart Study, which showed a significant association with blood pressure. Studies in more inbred populations are needed to rule out associations with lower levels of heterozygosity; at the extreme, consanguinity, which maximally suppresses heterozygosity, has been associated with CHD.[24] Equally, studies in populations in other ethnicities are warranted, as populations of African descent in particular are apt to exhibit variability at substantially larger numbers of loci.

In conclusion, in two cohort studies, genome-wide measures of heterozygosity were not associated with risk of incident CHD nor with CHD risk factors. If generalized heterozygosity provides any fitness advantage in humans, it seems unlikely to do so for chronic disease phenotypes like CHD at the levels of outbreeding seen in populations like these.
